# Continuous positive airway pressure in delivery room in extremely preterm infants: A single‐center retrospective study in China

**DOI:** 10.1002/pdi3.2507

**Published:** 2024-10-25

**Authors:** Xiaoting Zhang, Long Chen, Xiaoyun Zhong, Jiangfeng Ou, Yuan Shi

**Affiliations:** ^1^ Department of Neonatology Children's Hospital of Chongqing Medical University National Clinical Research Center for Child Health and Disorders Ministry of Education Key Laboratory of Child Development and Disorders China International Science and Technology Cooperation Base of Child Development and Critical Disorders Chongqing Key Laboratory of Pediatrics Chongqing China; ^2^ Department of Pediatrics Women and Children's Hospital of Chongqing Medical University Chongqing China

**Keywords:** continuous positive airway pressure, delivery room, extremely preterm infants

## Abstract

To assess the beneficial effects of delivery room continuous positive airway pressure (DRCPAP) in extremely preterm infants, a single‐center retrospective study was performed at the Women and Children's Hospital of Chongqing Medical University in China. Infants born between January 2016 and December 2018 were regarded as the control group, and those born between January 2019 and August 2022 were considered as the observation group (DRCPAP group). The primary outcome was tracheal intubation within 72 h after birth. Six hundred and seven patients were included in the study (control: 232; DRCPAP: 375). Compared with the control group, DRCPAP reduced the intubation rate (56.8% vs. 62.9%, OR 0.57, 95% CI 0.34–0.96, *p =* 0.035), including <28 weeks gestational age (GA) subgroup (61.5% vs. 84.7%, OR 0.12, 95% CI 0.02–0.78, *p =* 0.027). One‐to‐one propensity score matching (195:195) was used to match the baseline characteristics of patients in DRCPAP and control group. After matching, no significant differences were observed in intubation rate within 72 h between the two groups (20.5% [40 of 195] vs. 22.1% [43 of 195]; *p* = 0.711). Whether DRCPAP can reduce intubation rate within 72 h requires further investigation.

## Graphical abstract

In extremely preterm infants, nasal continuous positive airway pressure in the delivery room is recommended at the beginning of respiratory support to maintain functional residual volume, improve lung compliance, and increase oxygenation. However, when used in developing nations, these high‐quality studies from developed nations revealed some different results, such as failing to reduce the rate of mechanical ventilation and having a higher risk of pneumothorax. Also, no literature reports of large samples were reported in China, and we carried out this single‐center retrospective investigation to compare the short‐term outcomes of extremely preterm infants before and after the introduction of DRCPAP. To assess the beneficial effects of delivery room continuous positive airway pressure (DRCPAP) in extremely preterm infants.

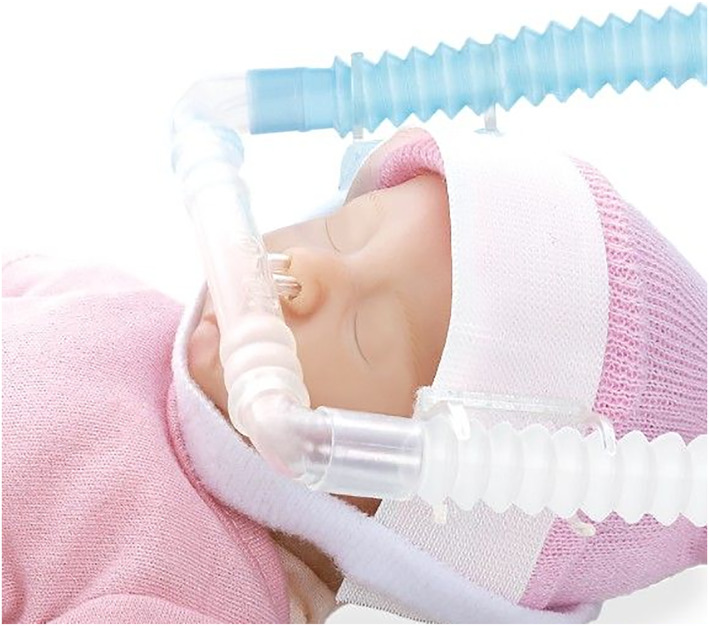

## 1 INTRODUCTION

The majority of extremely preterm infants struggle to establish functional residual volume and might frequently benefit from respiratory support.^1^ Due to their structural immaturity, lack of surfactant, and solid chest wall, the lungs of extremely preterm infants are especially prone to damage.^2^ In a randomized, multicenter experiment, infants who got continuous positive airway pressure (CPAP) treatment required intubation less frequently than infants who received surfactant treatment (within 1 h after birth).^3^ As a result, there are increased recommendations for extremely preterm infants to receive delivery room CPAP (DRCPAP).^4^, ^5^ In extremely preterm infants, DRCPAP is recommended at the beginning of respiratory support to maintain functional residual volume, improve lung compliance, and increase oxygenation.^6^

These findings have led to an increase in the number of extremely preterm infants stabilizing on DRCPAP.^7^ However, in certain underdeveloped regions, DRCPAP is not consistently utilized. When used in developing nations, these high‐quality studies from developed nations revealed some different results, such as failing to reduce the rate of mechanical ventilation (MV) and having a higher risk of pneumothorax.^8^ Also, no literature of large samples were reported in China, and we carried out this single‐center retrospective investigation to compare the short‐term outcomes of extremely preterm infants before and after the introduction of DRCPAP.

## 2 METHODS

In the Women and Children's Hospital of Chongqing Medical University, a retrospective study was carried out with a control group. All information was gathered using historical hospital records. Informed consent was obtained from parents or guardians. The study was approved by the Ethics Committee of the Chongqing Health Center for Women and Children (No. 2023003) and registered at www.chictr.org.cn (No.ChiCTR2300070366).

### 2.1 Inclusion and exclusion criteria

The study covered all premature babies with gestational age (GA) under 32 weeks.

Newborns with visible malformations or genetic syndromes, newborns delivered in different hospitals, at home, or elsewhere, newborns who died of congenital diseases after birth, and newborns with inadequate medical data were excluded.

### 2.2 Observation group (DRCPAP group)

Infants born between January 2016 and August 2022 were searched, and the observation group consisted of preterm infants born between January 2019 and August 2022.

According to the protocol for our unit, the initial DRCPAP was 6 cm H_2_O, the initial FiO_2_ was 0.30 for infants born before 28 weeks' GA and 0.21–0.30 for those born between 28 and 31 weeks. Positive pressure ventilation (PPV) was utilized with beginning peak inspiratory pressures of 20 cm H_2_O and positive end‐expiratory pressure of 6 cm H_2_O, respectively, for newborns who were persistently apneic or bradycardic (100 bpm). In the event that intubation was not necessary, the pulmonary surfactant (PS) was administered in the Delivery room (DR) using the less invasive surfactant administration (LISA) technique.

### 2.3 Control group

The control group consisted of infants born between January 2016 and December 2018 before DRCPAP was implemented. When apnea or significant respiratory distress was evident, endotracheal intubation was performed. After conducting the procedures with only oxygen inhalation or intubation via PPV (self‐inflating bag) and oxygen, the infants were immediately taken to the neonatal intensive care unit (NICU).

### 2.4 DR and NICU management

The infants in both groups received resuscitation according to the guideline procedures in the DR.^6^ LISA or intubate‐surfactant‐extubate (INSURE) technique would be taken into consideration in the NICU if the infant had severe respiratory distress syndrome (RDS).^9^ The initial dosage of surfactant was 200 mg/kg (Curosurf), or Calf PS was 100 mg/kg (Chian). All infants received non‐invasive or invasive respiratory support and caffeinated therapy as suggested by the guidelines.^9^, ^10^

After June 2018, several measures were gradually implemented. These included establishing a separate resuscitation room in the DR, maintaining an ambient temperature of 25°C–30°C, and practicing delayed cord clamping (DCC). A Giraffe incubator (Giraffe Incubator Carestation SC1, Ohmeda Medical) and Giraffe shuttle (Giraffe shuttle, Ohmeda Medical) were used for resuscitation/transition and intra‐hospital transfers.

### 2.5 Primary and secondary outcomes

The primary outcome was tracheal intubation within 72 h after birth.

The secondary outcomes were the other outcomes, including bronchopulmonary dysplasia (BPD), pneumothorax, extrauterine growth restriction (EUGR), necrotizing enterocolitis (NEC), intraventricular hemorrhage (IVH), time of oxygen therapy, and blood pressure after admission to NICU. The secondary outcome observation times are based on the time of diagnosis as per medical routine.

### 2.6 Data collection

Clinical information, such as respiratory support, surfactant therapy, days of invasive MV and oxygen therapy, BPD status, and corrected GA at discharge, were extracted from the infants' electronic medical records. Gender, birth weight, GA, maternal age, and prenatal steroid status were gathered among the demographic data.

### 2.7 Sample size calculation

This was a retrospective case study, divided into a DRCPAP group and a control group, with the rate of tracheal intubation as the main outcome indicator observed in the study population, which according to a review of the literature, was 48% vs. 28.2%^11^ in the two groups respectively. A bilateral *α* = 0.05 was set and the degree of certainty was 90%.

Using the PASS 2008 v8.0.3 (NCSS, Kaysville, Utah, USA), the sample size for the two groups was calculated to be *N* = 125 cases, and taking into account the lost to follow‐up situation of 20% calculation, the final minimum sample size subjects required were 156 cases, for a total of at least 312 study subjects that were included.

### 2.8 Statistical analysis

Continuous variables were compared using Student's *t*‐test or the Mann–Whitney rank sum test as appropriate. Categorical variables were compared using the *χ* ^2^ test. *p* values <0.05 were considered statistically significant.

We analyzed the baseline data comparing the control group with the DRCPAP group, and then a propensity score analysis with 1:1 matching was performed. The propensity score matching (PSM) was estimated using a logistic regression model in which the following covariates were included: GA, sex, birth weight, age of mother, age of father, vaginal delivery, singleton, perinatal factors (in vitro fertilization [IVF], premature rupture of membranes [PROM], gestational diabetes mellitus [GDM], intrahepatic cholestasis of pregnancy, clinical chorioamnionitis, prenatal glucocorticoid, hypertension), RDS (moderate and severe), and early onset sepsis (EOS). Those covariates were selected based on the previous literature.^8^ A 1:1 “nearest neighbor” case‐control match without replacement was used. Each neonate who received DRCPAP was matched with the control and the neonate who had the closest estimated propensity scores. Post‐match variables were compared between the groups using univariate analysis.

We conducted a logistic regression analysis of the primary outcome “tracheal intubation within 72 h”. First, the epidemiological data of the enrolled population were univariately analyzed between intubation and control groups. Then the variables with a *p* < 0.20 were entered in a stepwise binary logistic regression model after the selected predictor variables were checked for multicollinearity. The analysis of multicollinearity was performed, considering the condition index of eigenvalues and variables inserted in the model, and had to carry a variance inflation factor of <2. When the variables were correlated, the variable with the highest association with intubation was retained. The stepwise probability was 0.05 for entry and 0.1 for removal. Results were presented as odds ratio (OR) and 95% confidence interval (CI). In the subgroup analyses, the subjects were babies with GA under 28 weeks. Analyses were performed with SPSS 22.0.

### 2.9 Patient and public involvement

Patients or the public were not involved in the design, conduct, reporting, or dissemination plans of our research.

## 3 RESULTS

### 3.1 Participants

**FIGURE 1:**
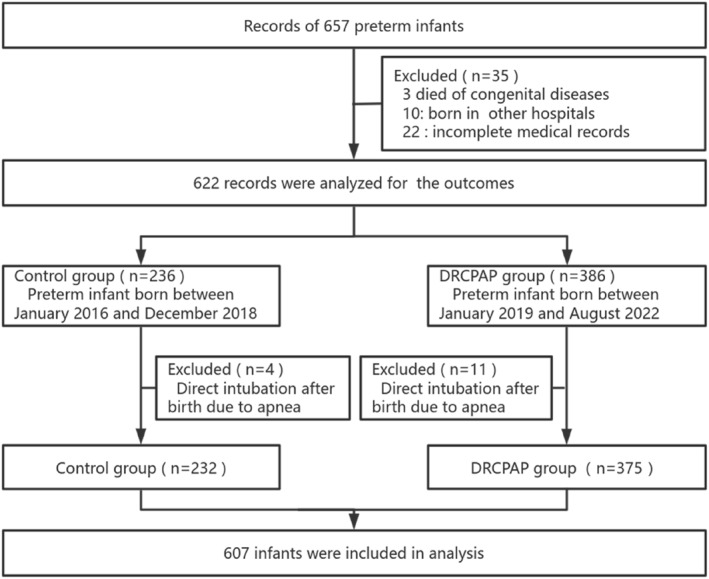
Flow chart of studied neonates.

Out of the 657 babies whose data were analyzed, 35 had incomplete records, including 10 babies born in other hospitals, 3 died of congenital diseases after birth, and 22 with incomplete medical records. We excluded 15 infants (control: 4, DRCPAP: 11) because they underwent immediate tracheal intubation after birth. As a result, 607 records (control: 232; DRCPAP: 375) were examined. The study flowchart is displayed in Figure 1.

The characteristics of the two groups before and after matching were shown in Table 1. Before matching, five out of the 17 covariates, including birth weight, vaginal delivery, IVF, RDS and antenatal corticoids were significantly different between the two groups. After matching, 390 cases were included in the PSM model. All 17 covariates were well balanced, and no significant differences were observed (Table 1).

**TABLE 1:** Clinical characteristics and outcomes between DRCPAP group and control groups.

	Before matching					After matching				
Clinical features	Total *n* = 607	DRCPAP group *n* = 375	Control group *n* = 232	OR (95% CI)	*p*‐value	Total *n* = 390	DRCPAP group *n* = 195	Control group *n* = 195	OR (95% CI)	*p*‐value
Gestational age (weeks) (IQR)	30.1 (28.4–31.1)	30.0 (28.3–31.1)	30.2 (28.7–31.2)	‐	0.080	30.2 (28.6–31.2)	30.2 (28.5–31.2)	30.1 (28.6–31.1)	‐	0.628
Birth weight (gram) (IQR)	1370 (1140–1600)	1300 (1070–15400)	1445 (1202–1620)	‐	<0.001	1405 (11,800–1612)	1440 (1150–1640)	1400 (1190–1600)	‐	0.810
Age of mother (IQR)	30.0 (28.0–34.0)	31.0 (2.08–34.0)	30.0 (28.0–32.0)	‐	0.142	30.0 (28.0–33.0)	31.0 (28.0–34.0)	30.0 (28.0–32.0)	‐	0.428
Age of father (IQR)	32.0 (29.0–36.0)	32.0 (29.0–36.0)	32.0 (29.0–36.0)	‐	0.891	32.0 (29.8–36.0)	33.0 (30.0–36.0)	32.0 (29.0–36.0)	‐	0.328
Vaginal delivery, *n*/%	131/21.6	55/14.7	76/32.8	0.35 (0.23–0.52)	<0.001	97/24.9	47/24.1	50/25.6	0.92 (0.58–1.45)	0.725
Singleton, *n*/%	360/59.3	212/56.5	148/63.8	0.73 (0.52–1.03)	0.077	230/59.0	112/57.4	118/60.5	0.88 (0.58–1.31)	0.537
Female, *n*/%	320/52.7	200/53.3	120/51.7	1.06 (0.76–1.48)	0.700	204/52.3	104/53.3	100/51.3	1.08 (0.73–1.61)	0.685
IVF, *n*/%	208/34.3	142/37.9	66/28.4	1.53 (1.07–2.18)	0.018	128/32.8	68/34.9	60/30.8	1.20 (0.78–1.84)	0.388
PROM, *n*/%	210/34.6	129/34.4	81/34.9	0.97 (0.69–1.37)	0.897	134/34.4	65/33.3	69/35.4	0.91 (0.60–1.38)	0.670
Clinical chorioamnionitis, *n*/%	35/5.8	25/6.7	10/4.3	1.58 (0.74–3.36)	0.226	16/4.1	7/3.6	9/4.6	0.77 (0.28–2.10)	0.610
GDM, *n*/%	166/27.3	110/29.3	56/24.1	1.30 (0.89–1.89)	0.163	101/25.9	48/24.6	53/27.2	0.87 (0.55–1.37)	0.563
Antenatal corticoids, *n*/%	386/63.6	259/69.1	127/54.7	1.84 (1.31–2.59)	<0.001	229/58.7	115/59.0	114/58.5	1.02 (0.68–1.52)	0.918
Hypertension, *n*/%	66/10.9	46/12.3	20/8.6	1.48 (0.85–2.57)	0.161	37/9.5	20/10.3	17/8.7	1.19 (0.60–2.36)	0.604
ICP, *n*/%	18/3.0	12/3.2	6/2.6	1.24 (0.46–3.36)	0.665	10/2.6	5/2.6	5/2.6	1.00 (0.28–3.51)	1.000
RDS, *n*/%	419/69.0	277/73.9	142/61.2	1.79 (1.26–2.54)	0.001	254/65.1	129/66.2	125/64.1	1.09 (0.72–1.66)	0.671
RDS ≥ 2 grades *n*, *n*/%	83/13.7	48/12.8	35/15.1	0.82 (0.51–1.32)	0.426	59/15.1	33/16.9	26/13.3	1.32 (0.75–2.31)	0.323
EOS, *n*/%	92/15.2	55/14.7	37/15.9	0.90 (0.57–1.42)	0.669	67/17.2	33/16.9	34/17.4	0.96 (0.57–1.63)	0.893
Intubation during 72 h, *n*/%	111/18.3	63/16.8	48/20.7	1.29 (0.85–1.96)	0.228	83/21.3	40/20.5	43/22.1	1.09 (0.67–1.78)	0.711
Apgar score at 1 min(IQR)	9.0 (8.0–10.0)	9.0 (8.0–10.0)	9.0 (8.0–10.0)	‐	0.179	9.0 (8.0–10.0)	9.0 (8.0–10.0)	9.0 (8.0–10.0)	‐	0.434
Apgar score at 5 min (IQR)	9.0 (10.0–10.0)	10.0 (9.0–10.0)	10.0 (9.0–10.0)	‐	0.230	10.0 (9.0–10.0)	10.0 (9.0–10.0)	10.0 (9.0–10.0)	‐	0.184
Surfactant (DR and NICU), *n*/%	312/51.4	194/51.7	118/50.9	1.03 (0.74–1.43)	0.835	201/51.5	92/47.2	109/55.9	0.70 (0.47–1.05)	0.085
DCC or UCM, *n*/%	347/57.2	325/86.7	22/9.5	62.04 (36.49–105.47)	<0.001	182/46.7	166/85.1	16/8.2	64.03 (35.56–122.16)	0.000
Time of transit from DR to NICU(IQR),min	22.0 (17.0–27.0)	24.0 (20.0–29.0)	19.0 (15.0–24.0)	‐	<0.001	21.0 (16.0–26.3)	23.0 (18.0–29.0)	19.0 (14.0–24.0)	‐	<0.001
Admission to PH(IQR)	7.26 (7.22–7.31)	7.26 (7.21–7.31)	7.27 (7.22–7.32)	‐	0.146	7.26 (7.21–7.32)	7.26 (7.21–7.31)	7.27 (7.21–7.32)	‐	0.458
Blood pressure after admission to NICU(IQR)	41.0 (35.0–45.0)	40.0 (35.0–45.0)	42.5 (35.0–47.0)	‐	0.007	42.0 (35.0–46.0)	41.0 (35.0–45.0)	42.0 (35.0–47.0)	‐	0.234
BPD, *n*/%	215/35.4	154/41.1	61/26.3	1.95 (1.36–2.79)	<0.001	123/31.5	68/34.9	55/28.2	1.36 (0.88–2.09)	0.157
BPD ≥ 2 grades, *n*/%	63/10.4	48/12.8	15/6.5	2.21 (1.16–3.88)	0.013	35/9.0	21/10.8	14/7.2	1.56 (0.76–3.16)	0.215
Pneumothorax, *n*/%	13/2.1	4/1.1	9/3.9	0.26 (0.08–0.87)	0.020	9/2.3	2/1.0	7/3.6	0.27 (0.05–1.35)	0.092
EUGR, *n*/%	240/39.5	142/37.9	98/42.2	0.83 (0.59–1.16)	0.284	152/39.0	65/33.3	87/44.6	0.62 (0.41–0.93)	0.022
NEC, *n*/%	49/8.1	41/10.9	8/3.4	3.43 (1.58–7.47)	0.001	29/7.4	22/11.3	7/3.6	3.41 (1.42–8.19)	0.004
IVH, *n*/%	113/18.6	71/18.9	42/18.1	1.05 (0.69–1.61)	0.799	80/20.5	45/23.1	35/17.9	1.37 (0.83–2.24)	0.210
Time of oxygen therapy (IQR)	16.1 (4.6–37.8)	22.7 (7.1–42.4)	5.5 (2.0–29.8)	‐	<0.001	12.3 (3.9–34.8)	20.0 (6.2–36.7)	5.9 (2.3–30.6)	‐	<0.001

Abbreviations: BPD, bronchopulmonary dysplasia; CI, confidence interval; DCC, delayed cord clamping; DR, delivery room; EOS, early onset sepsis; EUGR, extrauterine growth restriction; GDM, gestational diabetes mellitus; ICP, intrahepatic cholestasis of pregnancy; IQR, interquartile range; IVF, in vitro fertilization; IVH, intraventricular hemorrhage; NEC, necrotizing enterocolitis; NICU, neonatal intensive care unit; NRDS, neonatal respiratory distress syndrome; OR, odds ratio; PH, hydrogen‐ion concentration; PROM, premature rupture of membranes; SD, standard deviation; UCM, umbilical cord milking.

### 3.2 Primary outcome

Before matching, the overall intubation rate during 72 h was 18.3% (111 of 607), and after matching, the rate was 21.3% (83 of 390). There were no significant differences between the two groups (20.5% [40 of 195] vs. 22.1% [43 of 195]; *p* = 0.711; Table 1).

We conducted a logistic regression analysis of the primary outcomes “tracheal intubation within 72 h”. These variables were considered to have univariable associations with intubation within 72 h including GA, birth weight, Apgar 1 min, Apgar 5 min, EOS, PROM, GDM, antenatal corticoids, and surfactant, with a *p* value <0.2 (Table 2). And DRCPAP was also included.

**TABLE 2:** Clinical characteristics and outcomes between intubation and control groups.

Clinical features	Total (<32 weeks) *n* = 607	Intubation group *n* = 111	Control group *n* = 496	Or (95% CI)	*p*‐value	Total (<28 weeks)*n* = 98	Intubation group *n* = 39	Control group *n* = 59	Or (95% CI)	*p*‐value
Gestational age (weeks) (IQR)	30.1 (28.4–31.1)	28.5 (27.3–30.1)	30.2 (29.0–31.2)	‐	<0.001	27.1 (26.1–27.4)	27.1 (26.3–27.4)	27.1 (26.0–27.4)	‐	0.754
Birth weight (gram) (IQR or mean ± SD)	1370 (1140–1600)	1150 (950–1380)	1405 (1200–1620)	‐	<0.001	957 ± 132	950 ± 139	963 ± 128	‐	0.639
Apgar score at 1 min (IQR)	9.0 (8.0–10.0)	7.0 (5.0–8.0)	9.0 (8.0–10.0)	‐	<0.001	8.0 (6.0–9.0)	6.0 (5.0–8.0)	9.0 (8.0–9.0)	‐	<0.001
Apgar score at 5 min (IQR)	10.0 (9.0–10.0)	9.0 (8.0–9.0)	10.0 (9.0–10.0)	‐	<0.001	9.0 (8.0–10.0)	9.0 (6.0–9.0)	9.0 (8.0–10.0)	‐	<0.001
Age of mother (IQR)	30.0 (28.0–34.0)	30.0 (28.0–33.0)	30.0 (28.0–34.0)	‐	0.654	30.0 (27.8–32.0)	30.0 (28.0–31.0)	30.0 (27.0–33.0)	‐	0.844
Age of father (IQR or mean ± SD)	32.0 (29.0–36.0)	32.0 (29.0–36.0)	32.0 (29.0–36.0)	‐	0.833	32.3 ± 4.6	32.2 ± 4.4	32.4 ± 4.9	‐	0.899
Vaginal delivery, *n*/%	131/21.6	28/25.2	102/20.6	0.77 (0.48–1.25)	0.302	37/37.8	19/48.7	18/30.5	0.46 (0.20–1.06)	0.069
Singleton, *n*/%	360/59.3	64/57.7	296/59.7	1.08 (0.71–1.64)	0.695	45/45.9	16/41.0	29/49.2	1.39 (0.61–3.14)	0.429
Female, *n*/%	320/52.7	55/49.5	265/53.4	1.16 (0.77–1.76)	0.459	46/46.9	20/51.3	26/44.1	0.74 (0.33–1.68)	0.484
IVF, *n*/%	208/34.3	39/35.1	169/34.1	0.95 (0.62–1.46)	0.831	50/51.0	18/46.2	32/54.2	1.38 (0.61–3.11)	0.433
PROM, *n*/%	210/34.6	31/27.9	179/36.1	1.45 (0.92–2.29)	0.102	34/34.7	9/23.1	25/42.4	2.45 (0.99–6.06)	0.049
Clinical chorioamnionitis, *n*/%	35/5.8	6/5.4	29/5.8	1.08 (0.44–2.68)	0.857	4/4.1	1/2.6	3/5.1	2.03 (0.20–20.31_	0.924
GDM, *n*/%	166/27.3	23/20.7	143/28.8	1.55 (0.94–2.55)	0.083	33/33.7	7/17.9	26/44.1	3.60 (1.37–9.46)	0.007
Antenatal corticoids, *n*/%	386/63.6	64/57.7	322/64.9	1.35(0.89–2.06)	0.151	69/70.4	23/59.0	46/78.0	2.46 (1.01–5.97)	0.044
Hypertension, *n*/%	66/10.9	14/12.6	52/10.5	0.81 (0.43–1.52)	0.515	5/5.1	4/10.3	1/1.7	0.15 (0.01–1.40)	0.059
ICP, *n*/%	18/3.0	1/0.9	17/3.4	3.90 (0.51–29.64)	0.267	98/100	39/100	59/100	‐	
DRCPAP, *n*/%	375/61.8	63/56.8	312/62.9	1.29 (0.85–1.96)	0.228	74/75.5	24/61.5	50/84.7	3.47 (1.33–9.06)	0.009
Surfactant (DR and NICU) *n*/%	312/51.4	92/82.9	220/44.4	0.16 (0.09–0.27)	<0.001	91/92.9	37/94.9	54/91.5	0.58 (0.10–3.17)	0.529
DCC or UCM, delivery room *n*/%	347/57.2	55/49.5	292/58.9	1.45 (0.96–2.20)	0.073	66/67.3	21/53.8	45/76.3	2.75 (1.15–6.57)	0.020
EOS, *n*/%	92/15.2	32/28.8	60/12.1	0.34 (0.20–0.55)	<0.001	16/16.3	10/25.6	6/10.2	0.32 (0.10–0.99)	0.043
BPD, *n*/%	215/35.4	69/62.2	146/29.4	0.25 (0.16–0.39)	<0.001	89/90.8	35/89.7	54/91.5	1.23 (0.31–4.91)	0.765
BPD ≥ 2 grades *n*/%	63/10.4	28/25.2	35/7.1	0.22 (0.13–0.39)	<0.001	30/30.6	14/35.9	16/27.1	0.66 (0.27–1.58)	0.356
RDS, *n*/%	419/69.0	100/90.1	319/64.3	0.19 (0.10–0.37)	<0.001	95/96.9	38/97.4	57/96.6	0.75 (0.06–8.56)	0.816
RDS ≥ 2 grades *n*, *n*/%	83/13.7	32/28.8	51/10.3	0.28 (0.17–0.46)	<0.001	26/26.5	13/33.3	13/22.2	0.56 (0.22–1.40)	0.215
Pneumothorax, *n*/%	13/2.1	9/8.1	4/0.8	0.09 (0.02–0.30)	<0.001	3/3.1	2/5.1	1/1.7	0.31 (0.02–3.64)	0.714
EUGR, *n*/%	240/39.5	54/48.6	186/37.5	0.63 (0.41–0.95)	0.030	47/48.0	24/61.5	23/39.0	0.39 (0.17–0.91)	0.029
NEC, *n*/%	49/8.1	5/4.5	44/8.9	2.06 (0.79–5.33)	0.127	7/7.1	2/5.1	5/8.5	1.71 (0.31–9.30)	0.819
IVH, *n*/%	113/18.6	27/24.3	86/17.3	0.65 (0.39–1.06)	0.087	26/26.5	12/30.8	14/23.7	0.70 (0.28–1.73)	0.440
Time of oxygen therapy (IQR or mean ± SD)	16.0 (4.6–37.8)	38.3 (17.6–55.6)	12.8 (3.9–31.7)	‐	<0.001	55.5 ± 20.3	56.4 ± 20.6	54.8 ± 20.3	‐	0.707
Blood pressure after admission to NICU(IQR)	41.0 (35.0–45.0)	38.0 (32.0–44.0)	42.0 (35.0–46.0)	‐	<0.001	35.0 (32.0–40.0)	33.0 (31.0–38.0)	35.0 (32.0–42.0)	‐	0.106
Time of transit from DR to NICU(IQR),min	22.0 (17.0–27.0)	24.0 (19.0–30.0)	22.0 (17.0–27.0)	‐	0.004	25.5 (21.0–30.3)	25.0 (191.0–31.0)	26.0 (22.0–30.0)	‐	0.711

Abbreviations: BPD, bronchopulmonary dysplasia; CI, confidence interval; DCC, delayed cord clamping; DR, delivery room; EOS, early onset sepsis; EUGR, extrauterine growth restriction; GDM, gestational diabetes mellitus; ICP, intrahepatic cholestasis of pregnancy; IQR, interquartile range; IVF, in vitro fertilization; IVH, intraventricular hemorrhage; NEC, necrotizing enterocolitis; NICU, neonatal intensive care unit; NRDS, neonatal respiratory distress syndrome; OR, odds ratio; PH, hydrogen‐ion concentration; PROM, premature rupture of membranes; SD, standard deviation; UCM, umbilical cord milking.

DRCPAP, surfactant, GA, PROM, GDM, antenatal corticoids, EOS, and Apgar score at 1 min were included in the final multivariable model. In the binary logistic regression analysis, variables that retained independent associations with intubation during 72 h were DRCPAP (OR 0.57, 95% CI 0.34–0.96, *p* = 0.035), surfactant (OR 3.60, 95% CI 1.84–7.01, *p* < 0.001), GA (OR 0.78, 95% CI 0.67–0.92, *p* = 0.003), EOS (OR 2.59, 95% CI 1.43–4.69, *p* = 0.002), and Apgar 1 min (OR 0.59, 95% CI 0.52–0.6, *p* < 0.001) (Table 3).

**TABLE 3:** Independent risk factors for intubation during 72 h.

<32 weeks	OR	95% CI	*p*‐value
DRCPAP	0.57	0.34–0.96	0.035
Surfactant (DR and NICU)	3.60	1.84–7.01	<0.001
Gestational age	0.78	0.67–0.92	0.003
PROM	1.16	0.67–2.02	0.585
GDM	0.57	0.31–1.03	0.067
Antenatal corticoids	1.07	0.62–1.85	0.791
EOS	2.59	1.43–4.69	0.002
Apgar score at 1 min	0.59	0.52–0.68	<0.001
**<28 weeks**	**OR**	**95% CI**	***p*** **‐value**
DRCPAP	0.12	0.02–0.78	0.027
PROM	0.78	0.22–2.73	0.704
GDM	0.29	0.08–1.08	0.065
Antenatal corticoids	1.22	0.30–4.95	0.772
Hypertension	7.50	0.46–122.55	0.157
Blood pressure after admission to NICU	0.95	0.87–1.04	0.281
Vaginal delivery	1.02	0.26–4.01	0.968
DCC or UCM	2.40	0.40–14.45	0.336
EOS	4.48	1.06–18.95	0.041
Apgar score at 1 min	0.48	0.34–0.69	<0.001

Abbreviations: CI, confidence interval; DR, delivery room; DRCPAP, delivery room continuous positive airway pressure; EOS, early onset sepsis; GDM, gestational diabetes mellitus; Min, minute; NICU, neonatal intensive care unit; OR, odds ratio; PROM, premature rupture of membranes.

### 3.3 Secondary outcomes

BPD, EUGR, NEC, IVH, pneumothorax, time of oxygen therapy, and blood pressure after admission to NICU were shown in Table 1.

## 4 DISCUSSION

Our investigation was carried out in China and was a single‐center retrospective study. We evaluated the incidence of tracheal intubations performed upon extremely preterm infants during their first 72 h in the DR and NICU before and after using DRCPAP. According to our findings, compared with the control group, DRCPAP reduced the intubation rate (56.8% vs. 62.9%, OR 0.57, 95% CI 0.34–0.96, *p =* 0.035), including <28 weeks GA subgroup (61.5% vs. 84.7%, OR 0.12, 95% CI 0.02–0.78, *p =* 0.027; Table 3). Also, the findings indicated that in both groups, the probability of tracheal intubation was higher the lower the Apgar score at 1 min. The risk of tracheal intubation increased in neonates with early‐onset sepsis.

To date, several similar studies have been carried out, and our primary findings were in line with this earlier research.^3^, ^12^, ^13^ A randomized multicenter experiment^3^ involving 1316 infants (24 weeks 0 days and 27 weeks 6 days of gestation) comparing intubation and surfactant treatment (within 1 h after birth) with CPAP treatment initiated in the DR, showed that, compared with the surfactant, less frequently required intubation or postnatal corticosteroids for BPD (*p* < 0.001), required fewer days of MV (*p* = 0.03), and were more likely to be alive and free from the need for MV by day 7 (*p* = 0.01) in the CPAP group. Vieira et al.^12^ also indicated that there were significant reductions in intubation rate (89% vs. 73%, *p* = 0.02) after adopting an “early CPAP” protocol in preterm infants with a GA between 28 and 32 weeks in a retrospective study involving 109 infants. However, there are still different findings. A multicenter randomized clinical trial (RCT) by Gonçalves‐Ferri et al.^13^ involved 197 infants with a birth weight of 1000–1500 g who were not intubated or extubated within 15 min after birth and were randomized for routine or CPAP. The results reported that there were no statistically significant differences in the need for MV during the first 5 days of life (19.2 vs. 23.4%, *p* = 0.50), use of surfactant (18.2 vs. 17.3%, *p* = 0.92), or respiratory morbidity and mortality until discharge between the two groups.

One cause to explain the results might be the distance and the transport duration between DR and NICU room. Terrin et al.^14^ suggested that the DR and the NICU room should be directly interconnected to reduce morbidity in preterm neonates. A report by Trevisanuto et al.^15^ showed that the average transit times from the closest and furthest delivery rooms to the NICU were about 5 and 10 min. Kakkilaya et al.^16^ in an “Improvement Project to Decrease DR Intubations in Preterm Infants,” showed that the hospital relocated to a new larger facility in August 2015 where the DR was one floor below the NICU but did not mention the distance and transit time. Other articles on DRCPAP studies did not mention this aspect, but the cases in the single‐center study were all from the same hospital, both obstetrical and pediatric, presuming that the distances and times should be relatively short. In our study, in a women and children's hospital, the DR is one floor below the NICU, with direct access from the middle elevator. Our study showed that the average time was 22.0 (17.0–27.0) min, 24.0 (19.0–30.0) in the intubation group, and 21.0 (16.0–27.0) in the control group (*p* = 0.004). This time included the resuscitation stabilization time in the DR and the transit time. While there are statistical differences between the two groups, the actual time difference in the work is not significant from a clinical perspective. Further research is required.

Another important cause might be the differences between countries and regions. Desai et al.^17^ conducted a research in India that showed that nine neonates (30%) in the DRCPAP group required MV versus 26 neonates (86.67%) in the control group (*p* = 0.0001). Dunn et al.‘s^18^ study in Canada in 2011 showed that in the CPAP group, 48% were managed without intubation and ventilation, and 54% were without surfactant treatment. We hypothesized that the level of health care in the country and region might impact the outcome of DRCPAP.

The third cause might be the differences in observation time. In a randomized multicenter trial^3^ chose “the need for MV by day 7” in the study, and CPAP groups were more likely to be alive and free from the need for MV by day 7 (*p* = 0.01). The observation time of intubation or MV in the study by Gonçalves‐Ferri et al.^13^ was “in the first 5 days of life” and that was “between study enrollment and hospital discharge” by Tapia et al.^19^

Furthermore, the fourth cause to explain the inconsistency among different studies might be GA. A randomized multicenter trial^3^ in 2010 showed that in 24–27^+6^ weeks, the infants who received CPAP treatment, as compared with surfactant treatment, less frequently required intubation or postnatal corticosteroids for BPD (*p* < 0.001), and required fewer days of MV (*p* = 0.03). Grover et al.^20^ in 2022, indicated that in 28–36 weeks, there were no statistically significant differences between the treatment failure rates with a heated humidified high‐flow nasal cannula and CPAP (13.1% vs. 11.1%, 95% CI 9.9–14.07, *p* = 0.73). Abuel Hamd et al.^21^ in 2020, chose between 27 and 32 weeks and reported that there were no differences between the sustained lung inflation group and the control group (55% vs. 65%, OR 0.623, 95% CI 0.33–1.18, *p* = 0.145) in the outcomes of MV in the first 72 h of life.

There are drawbacks to this study. It was a retrospective study, and while there were clear guidelines for the clinical management of both DR and NICU, the intervention was not blinded, which left room for bias. Once PPV was started, clinicians in the historical control group may have been more inclined to intubate infants in the PPV group, which could represent a possible bias. Additionally, the control group was included in the study from 2016 to 2018, while the observation group was included after 2019. There are period differences between the two groups. Although the NICU treatment measures of the two groups are similar, there may still be additional confounding factors in neonatal treatment and resuscitation, such as setting up an independent resuscitation chamber, maintaining an ambient temperature of 25°C–30°C, and DCC. A Giraffe incubator (Giraffe Incubator Carestation SC1, Ohmeda Medical) and Giraffe shuttle (Giraffe shuttle, Ohmeda Medical) were used for resuscitation/transition and intra‐hospital transfers. These factors could potentially impact the study findings. Finally, between DRCPAP and control group, following the PSM, the tracheal intubation rate between the two groups is not different, but there is a reduction in data sample size after matching, which could introduce bias to the results. Given the retrospective design, there may be bias and confounding factors that necessitate making generalizations regarding safety or effectiveness in a prospective RCT.

## 5 CONCLUSION

Compared with control, DRCPAP lowers the incidence of 72‐h tracheal intubation.

## AUTHOR CONTRIBUTIONS

Dr. Xiaoting Zhang drafted the manuscript, participated in designing the study and was responsible for the inclusion and exclusion of studies, assessment of methodological quality, data analysis, writing, and revision. Dr. Long Chen participated in designing the study, interpreted the data, and edited the manuscript. Dr. Xiaoyun Zhong initiated and managed the study, recruited the participants, Dr. Jiangfeng Ou assisted in the data analysis and the editing of the manuscript. Dr. Yuan Shi conceptualized and designed the study, and critically reviewed the manuscript for important intellectual content. All the authors contributed to the revision of the manuscript and approved the final version.

## CONFLICT OF INTEREST STATEMENT

The authors have no conflicts of interest relevant to this article to disclose.

## ETHICS STATEMENT

The study was approved by the Ethics Committee of Women and Children’s Hospital of Chongqing Medical University (No. 2023003) and registered at www.chictr.org.cn (No.ChiCTR2300070366).

## Data Availability

The datasets generated during and/or analyzed during the current study are available from the corresponding author on reasonable request. The data that support the findings of this study are available from the corresponding author upon reasonable request.
